# Transcriptional profiling in response to terminal drought stress reveals differential responses along the wheat genome

**DOI:** 10.1186/1471-2164-10-279

**Published:** 2009-06-24

**Authors:** Alessio Aprile, Anna M Mastrangelo, Anna M De Leonardis, Gabor Galiba, Enrica Roncaglia, Francesco Ferrari, Luigi De Bellis, Luana Turchi, Giovanni Giuliano, Luigi Cattivelli

**Affiliations:** 1 CRA-Genomic Research Centre, Via S.Protaso 302, 29017 Fiorenzuola d'Arda, Piacenza, Italy; 2 Department of Science and Biological and Environmental Technologies, University of Salento, Provinciale Lecce-Monteroni, 73100 Lecce, Italy; 3 CRA-Cereal Research Centre, Strada Statale16, km 675, Foggia, Italy; 4 Agricultural Research Institute of the Hungarian Academy of Sciences, 2462 Martonvasar, Hungary; 5 University of Modena and Reggio Emilia, Department of Biomedical Sciences, Via Campi 287, 41100, Modena, Italy; 6 University of Padova, Department of Biology, Via G. Colombo 3, 35121, Padova, Italy; 7 ENEA-Casaccia Research Centre, Via Anguillarese 301, 00123 Roma, Italy

## Abstract

**Background:**

Water stress during grain filling has a marked effect on grain yield, leading to a reduced endosperm cell number and thus sink capacity to accumulate dry matter. The bread wheat cultivar Chinese Spring (CS), a Chinese Spring terminal deletion line (CS_5AL-10) and the durum wheat cultivar Creso were subjected to transcriptional profiling after exposure to mild and severe drought stress at the grain filling stage to find evidences of differential stress responses associated to different wheat genome regions.

**Results:**

The transcriptome analysis of Creso, CS and its deletion line revealed 8,552 non redundant probe sets with different expression levels, mainly due to the comparisons between the two species. The drought treatments modified the expression of 3,056 probe sets. Besides a set of genes showing a similar drought response in Creso and CS, cluster analysis revealed several drought response features that can be associated to the different genomic structure of Creso, CS and CS_5AL-10. Some drought-related genes were expressed at lower level (or not expressed) in Creso (which lacks the D genome) or in the CS_5AL-10 deletion line compared to CS. The chromosome location of a set of these genes was confirmed by PCR-based mapping on the D genome (or the 5AL-10 region). Many clusters were characterized by different level of expression in Creso, CS and CS_AL-10, suggesting that the different genome organization of the three genotypes may affect plant adaptation to stress. Clusters with similar expression trend were grouped and functional classified to mine the biological mean of their activation or repression. Genes involved in ABA, proline, glycine-betaine and sorbitol pathways were found up-regulated by drought stress. Furthermore, the enhanced expression of a set of transposons and retrotransposons was detected in CS_5AL-10.

**Conclusion:**

Bread and durum wheat genotypes were characterized by a different physiological reaction to water stress and by a substantially different molecular response. The genome organization accounted for differences in the expression level of hundreds of genes located on the D genome or controlled by regulators located on the D genome. When a genomic stress (deletion of a chromosomal region) was combined with low water availability, a molecular response based on the activation of transposons and retrotransposons was observed.

## Background

Drought stress greatly affects productivity and growth of plants and plays a central role in their geographical range. Water deprivation induces a set of physiological and biochemical responses in plants and is one of the most complex adverse conditions, since it depends not only on the severity and duration of the stress event, but also on the plant developmental stage and morphology [1,2].

Soon after the perception and recognition of external changes, different signaling pathways are activated in order to convert a physical stress into a biochemical response, each of them promoting the expression of a set of stress-responsive genes; the full activation of signal cascades induced by a given stress event promotes acclimation and leads to stress tolerance.

The main physiological drought stress responses include stomatal closure, repression of cell growth and photosynthesis, and activation of respiration. At the biochemical level, many plants accumulate osmoprotectants such as sugars (sucrose, raffinose, trehalose), sugar alcohols (sorbitol and mannitol), amino acids (proline), and amines (glycine betaine and polyamines) [2,3]. These metabolites also act as antioxidants or scavengers helping plants to avoid and/or tolerate stresses.

Drought stress triggers the production of the phytohormone abscisic acid (ABA). Several drought-inducible genes are induced by exogenous ABA treatment, whereas others are not affected, indicating the presence of both ABA-independent and ABA-dependent regulatory systems [4].

Many drought-inducible genes with various functions, including a number of transcription factors that regulate stress-inducible gene expression, have been identified by molecular and genomic analyses. Many families of plant transcription factors are involved in the stress-induced signaling cascade. Among them: bZIP proteins (ABRE-binding factors [5]), MYC, MYC-like, bHLH and MYB proteins [4,6], WRKY proteins [7], Cbf/DREB1 (C-repeat binding factor/dehydration-responsive element-binding factor1) and DREB2 [8,9]. Recently, a number of stress-inducible genes have been identified using microarray analysis in different plant species, such as Arabidopsis, rice, barley and grape [10-18]. Furthermore the ectopic expression of several stress induced genes with a key role in the stress response pathways has resulted in improved plant stress tolerance [19,20].

In wheat, by mean of special genetic stocks (i.e. single chromosome recombinant lines), several chromosomes or chromosome regions carrying major genes affecting environmental stress response were identified [21]. Genes affecting flowering time and abiotic stress responses in wheat are particularly concentrated on chromosomes belonging to group 5, especially on 5A [22-26]. Using wheat deletion stocks genes affecting frost tolerance and vernalization requirement [27], copper stress tolerance [28], traits affecting osmoregulation (carbohydrate, amino acid and polyamine content, reviewed in [29]) were physically mapped between deletion breakpoint 0.56 and the telomeric end of chromosome 5A.

The productivity of wheat, one of the most important crops worldwide, is often limited by shortage of water necessary to maximize biomass and complete grain filling [30]. In the present study we performed a transcriptional profiling of three wheat genotypes with different genome organization, under medium and severe drought stress conditions at the grain filling stage. We compared the hexaploid bread wheat cultivar Chinese Spring (CS, genome AABBDD) with a deletion line CS-5AL-10 carrying a chromosome deletion at the breakpoint 0.56, and with a tetraploid durum wheat (genome AABB) to find evidences of differential responses associated to different wheat genome regions.

## Results

### Physiological responses

To provide a global study of transcriptome changes in response to drought stress, a durum wheat (*Triticum durum *Desf. cultivar Creso) and two bread wheat (*Triticum aestivum *L. cultivar Chinese Spring -CS- and its deletion line CS_5AL-10) genotypes were subjected to two different levels of water stress at the grain filling stage in controlled conditions. Although the three genotypes were grown and exposed to drought in presence of the same amount of available water in the soil (28%, 18% and 12% SWC for control -CTRL-, mild stress -MS- and severe stress -SS-, respectively), their leaf water potentials were different (Table 1). The plants of the durum wheat cv. Creso were characterized by a more negative leaf water potential in all treatments; furthermore, they reached these values of water potential earlier than the bread wheat cv. CS (4 days *vs *6 days) suggesting that Creso underwent a faster water loss than CS, probably due to a delay in the activation of the water stress responsive mechanisms.

**Table 1:** Leaf water potential of Creso, CS and CS_5AL-10 subjected to water stress treatment

**Genotype**	**Treatment**	**Leaf water potential ψ_w _(MPa)**	**Soil Water Content (SWC)**
**Creso (durum wheat)**	*CTRL*	-1.4 ± 0.058	*28%*
	*MS*	-2.6 ± 0.173	*18%*
	*SS*	-3.9 ± 0.115	*12.5%*
**CS (bread wheat)**	*CTRL*	-1.2 ± 0.058	*28%*
	*MS*	-2.2 ± 0.058	*18%*
	*SS*	-3.3 ± 0.058	*12.5%*
**CS_5AL-10**	*CTRL*	-1.1 ± 0.058	*28%*
	*MS*	-2.1 ± 0.115	*18%*
	*SS*	-3.4 ± 0.173	*12.5%*

Soil water content and leaf water potential values corresponding to control (CTRL), moderate (MS) and severe stress (SS) in each genotype.

### Microarray quality analysis

GeneChip^® ^hybridization quality was verified using the standard Affymetrix controls. All hybridizations showed the expected checkerboard pictures. The average background was 39.96, well within the recommended levels. The percentage of "present" calls ranged between 42.35% and 51.78% among the 61 K probe sets present on the array. Durum wheat samples showed percentage of "presents" constantly lower than bread wheat samples, in agreement with their genome sizes. Pearson correlation coefficients computed on the RMA expression values (log2-transformed) for each set of biological triplicates ranged from 0.93 to 0.99.

**Figure 1:**
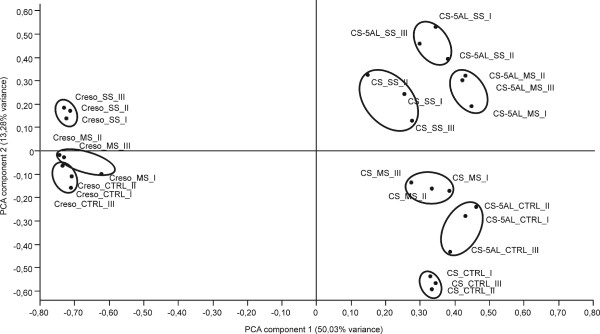
**PCA plot of the wheat array hybridization data**. The x and y axis represent the two principal components of the total variance, 50.03% and 13.28%, respectively. Each oval groups samples from the same genotype and treatment. CTRL = Control samples, MS = Moderate stress samples, SS = Severe stress samples.

Four main sources of variation explaining 75.43% of total variance were identified by principal component analysis (PCA). The two main components explain 50.03% and 13.28% of variance (Figure 1). Data represented on a scatter plot with the first and second components on the x and y axis, respectively, showed that the replicates of each sample clustered together, as expected. The samples belonging to bread wheat and durum wheat were completely separated on the x axis indicating that the main source of variation was due to the genotype factor. The second source of variation, along the y axis, was due to the treatment factor. CTRL, MS and SS samples of CS were well separated from each other; whereas the MS and SS samples in CS_5AL-10, and the CTRL and MS samples in Creso were poorly separated. This finding suggests that the extent of transcriptome remodeling in MS and SS conditions was different in the three genotypes. While in CS each level of drought was sufficient to induce a strong molecular response, in Creso the MS condition was not sufficient to induce a sizable response and only a minimal variation was observed in CS_5AL-10 between SS and MS conditions.

### Transcriptome changes

To identify subsets of genes differentially expressed in response to drought treatments or among genotypes, the array data were analyzed using a Welch t-test with Benjamini and Hochberg false discovery rate correction for multiple tests [31]. Fold-change was then employed as a surrogate measure of biological significance for gene selection [32].

As shown in Table 2, nine comparisons were done to identify drought stress-regulated genes. According with the definition given in Materials and Methods, 3,056 probe sets were found to be differentially expressed (see additional file 1: Differentially expressed genes under drought conditions). Three further comparisons were performed to identify genes differentially expressed among the considered genotypes and not involved in the drought response. The comparison among the CTRL samples of Creso, CS and CS_5AL-10 has identified 8,552 non redundant probe sets with a significantly different expression level.

**Table 2:** Summary of comparisons.

**Comparison**	**Probe sets >2X** **Up-regulated**	**Probe sets >2X** **Down-regulated**	**Total regulated probe sets**
**Creso**			

MS *Vs *CTRL	0	0	0
SS *Vs *MS	191	302	493
SS *Vs *CTRL	661	809	1470

**CS**			

MS *Vs *CTRL	54	52	106
SS *Vs *MS	29	0	29
SS *Vs *CTRL	579	263	842

**CS_5AL-10**			

MS *Vs *CTRL	287	106	393
SS *Vs *MS	47	0	47
SS *Vs *CTRL	660	527	1187

**CTRL**			

CS *Vs *Creso	3974	2662	6636
CS-5AL-10 *Vs *Creso	3889	3062	6951
CS-5AL-10 *Vs *CS	226	381	607

In the first column analysis comparisons are listed. Number of differentially expressed probe sets under drought with a minimun 2-fold change is reported. The comparisons between not stressed samples of CS, CS-5AL and Creso are also shown. CS = Chinese Spring, CS-5AL = Chinese Spring 5A deletion line, CTRL = control condition, MS = mild stress condition, SS = severe stress condition.

The comparison between MS and CTRL samples yielded 106 differentially expressed probe sets in CS and 393 in CS_5AL-10, whereas in Creso no significant differences were found. An opposite trend was noticed when SS samples were compared with MS ones: few changes in gene expression were observed in bread wheat samples (29 in CS and 47 in CS_5AL-10), whereas a set of 493 transcripts in SS Creso were significantly different from MS (Table 2). Finally, the comparisons between SS and CTRL samples yielded many more differentially expressed genes in Creso (1470) than in CS (842) (Table 2), a difference of more than six hundred probe sets.

Although the leaf water potential in MS plants was more negative in the durum wheat Creso than in the bread wheat CS (or CS_5AL-10), significant variations in mRNA levels in response to MS were detected only in the hexaploid genotypes.

The comparisons between the two bread wheat CTRL and the durum wheat CTRL samples gave about 6800 differentially expressed probe sets (Table 2), a result that is largely dependent on the presence of the D genome in hexaploid wheat. The comparison between CS_5AL-10 and CS showed 607 differentially expressed transcripts, probably due to the partial deletion of chromosome 5A.

Fifteen probe sets, representing genes putatively involved in the drought stress responses, were subjected to real-time qRT-PCR analysis to validate the array data. The poliubiquitin gene corresponding to the probe set Ta.24299.1.S1_at was selected based on its minimal coefficient of variation, and used as reference gene in qRT-PCR. Although the magnitude of the transcript expression was, to some extent, different between array and qRT-PCR, all tested genes showed the same expression trend with the two methods. The Pearson product-moment correlation coefficients between microarray and qRT-PCR data were 0.909***, 0.972*** and 0.870*** for Creso, CS and CS-5AL, respectively (*** P < 0.001).

### QT-Clustering

**Figure 2:**
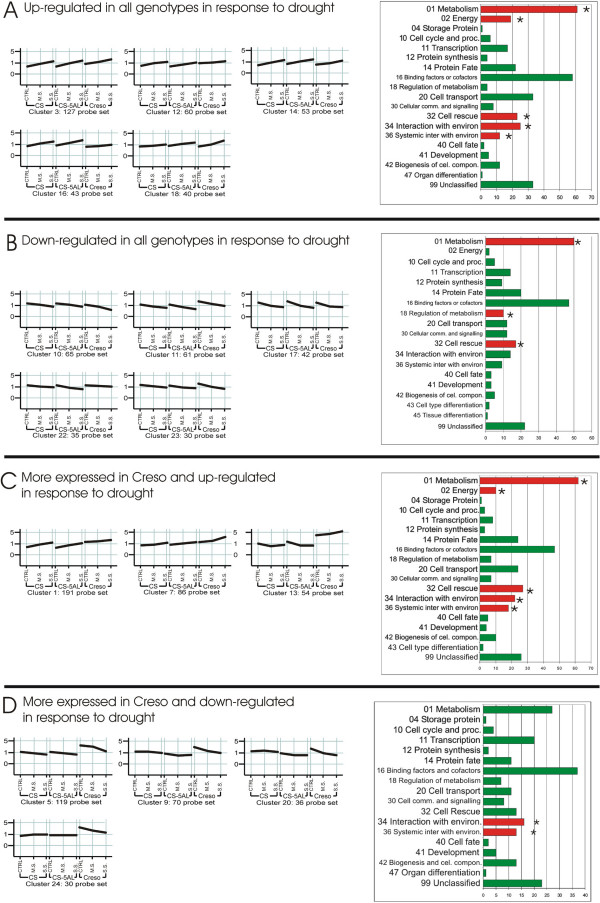
**Representation of 17 out of 24 QT-clusters obtained using the expression values of the 3,056 stress-related genes differentially expressed in at least one condition/genotype**. The cluster analyses was performed with a minimum cluster size of 30 and a correlation value of 0.75. The three treatments, grouped by genotypes, are plotted on x axis. The relative expression level (data normalized to the median for each probe set) is plotted on the y axis. The horizontal lines represent the average expression of all probe sets belonging to each cluster. 1,376 probe sets didn't fit the QT-clustering parameters.

**Figure 3:**
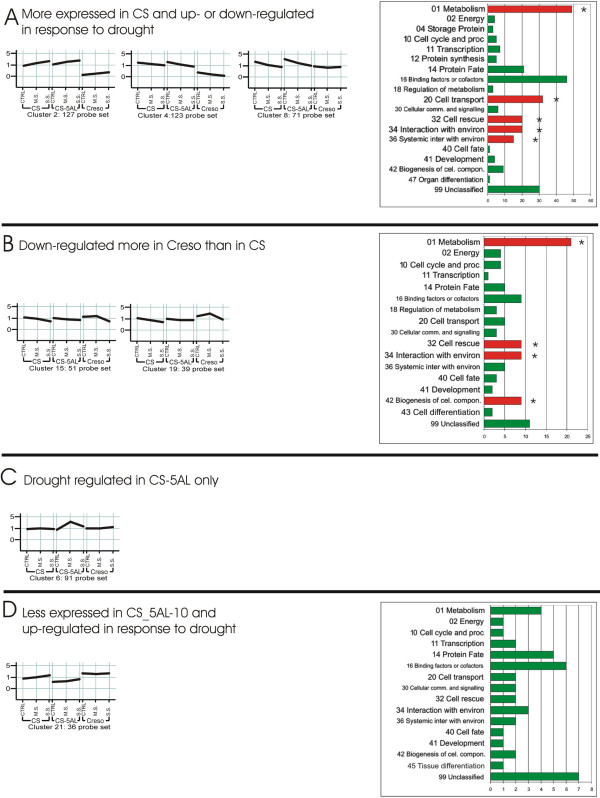
**Representation of 7 out of 24 QT-clusters obtained using the expression values of the 3,056 stress-related genes differentially expressed in at least one condition/genotype**. The cluster analyses was performed with a minimum cluster size of 30 and a correlation value of 0.75. The three treatments, grouped by genotypes, are plotted on x axis. The relative expression level (data normalized to the median for each probe set) is plotted on the y axis. The horizontal lines represent the average expression of all probe sets belonging to each cluster. 1,376 probe sets didn't fit the QT-clustering parameters.

As mentioned above, 3,056 probe sets were differentially expressed as a consequence of drought stress in at least one comparison (the list of all drought-regulated genes is presented in the additional file 1: Differentially expressed genes under drought conditions). QT-cluster analysis [33] was performed and interpreted to identify groups of genes whose expression can be associated to the different genome structures. Bioinformatic analysis (minimum cluster size 30, correlation value 0.75) yielded 24 clusters (Figure 2 and Figure 3) plus 1376 unclassified probe sets.

Ten clusters (5 up- and 5 down-regulated) grouped 556 probe sets responsive to drought to a similar extent in all three genotypes (Figure 2A and 2B). These genes represent the stress response mechanisms common to bread and durum wheat. The analysis with the MIPS FunCat tool [34] of the probe sets in the up-regulated clusters identified five main over-represented categories (0.005 p-value cut off), most of them related to water stress (Figure 2A). Among these categories, subcategories such as "01.01.03.03 Metabolism of proline", "32.01.03 Osmotic and salt stress response", "34.11.03.13 Osmosensing and response", "36.20.18.02 Ethylene response", "36.20.18.05 Abscisic acid response" were all over-represented. Many well known components of the molecular response to drought belong to these clusters. For instance, the expression levels of four 9-cis-epoxycarotenoid-dioxygenase (*NCED*)-related probe sets (cluster 3), the key enzyme of ABA biosynthesis [35], were strongly up-regulated by water stress to similar extent in all genotypes.

Clusters 3 and 14 are also characterized by the presence of additional stress-related signaling components and transcription factors (two kinase enzymes involved in signal transduction, eight MYB family-related, one WRKY-related, one DOF-related and three probe sets with a Zinc Finger motif) as well as of probe sets related to osmolyte pathways (sorbitol, glycine betaine and proline). The probe set Ta.21428.1.S1_x_at has high similarity to sorbitol dehydrogenase, the key enzyme in sorbitol accumulation [36]. Aldehyde dehydrogenases (ADH) are involved in aldehyde detoxification and the induction of genes encoding ADHs was often associated to drought stress [37]. Moreover, a substrate-specific aldehyde dehydrogenase (betaine aldehyde dehydrogenase, BADH) is responsible of glycine betaine biosynthesis [38]. Two ADH-related probe sets (Ta.18775.1.S1_at, Ta.25596.3.A1_a_at) and a BADH-related one (Ta .435.1.S1_at) were found in cluster 3 and 14. Several probe sets involved in proline accumulation were also classified in these clusters (detailed hereafter).

Overall, the up-regulated clusters illustrated in Figure 2A contain most of the known drought-responsive genes whose induction is conserved among all three genotypes and co-regulated with *NCED *expression, suggesting that a large proportion of these genes might be ABA-dependent. Notably, the *LEA *genes, one of the most studied family of drought-responsive sequences [39], were only marginally represented among the probe sets up-regulated in response to drought in wheat during grain filling. Among the 179 probe sets corresponding to *LEA *genes loaded on the Wheat Genome Array, only 16 were up-regulated in the experiment here described. Most of them (12) were unclassified after cluster analysis, indicating that their expression cannot be associated with the accumulation of the other well known drought-responsive genes above described.

Besides the expected drought-related induction/repression, many clusters showed an expression profile strongly dependent on the genome organization of the three genotypes analyzed. Seven clusters (Figure 2C and 2D) were characterized by a higher expression level in the CTRL of Creso compared with the corresponding sample of CS and CS_5AL-10. The functional classification of the 586 probe sets belonging to these seven clusters revealed five main categories over-represented: "01 Metabolism", "02 Energy", "32 Cell rescue defense and virulence", "34 Interaction with environment", "36 Systemic interaction with environment". The 191 probe sets of cluster 1 showed a constitutively high expression and a minimal drought induction in Creso, whereas their expression was proportional to the degree of stress in CS and CS_5AL-10. Analysis for functional categories revealed that "01.05 C-compound and carbohydrate metabolism", "01.20 Secondary metabolism", "02.45 Photosynthesis" and "02.45 Energy conversion and regeneration" were the subcategories over-represented. These results suggest that the probe sets belonging to cluster 1 are mainly involved in the adaptation of photosynthesis and carbohydrate metabolism to drought. In bread wheat the more severe the drought stress, the higher the expression level of these genes supporting their involvement in stress adaptation. This adaptation was not observed in Creso, where these mRNAs showed a constitutive high level of expression.

On the contrary, clusters 2, 4 and 8 contain probe sets whose expression levels were lower in Creso than in CS and CS_5AL-10 (Figure 3A). Part of them were not expressed at all in Creso; they can therefore be considered bread wheat-specific. Whereas the probe sets of cluster 2 were up-regulated by drought stress, clusters 4 and 8 genes were down-regulated. Cluster 2 contains many genes known to be involved in drought and osmotic stress response such as *RAB18 *dehydrin, aldose reductase (catalyse the reaction from glucose to sorbitol involved in the detoxification of aldehydes [40]), ornithine cyclodeaminase (involved in proline synthesis from ornithine [41]). The probe set Ta.10398.1.S1_at, encoding aldose reductase, was not expressed in Creso (expression values lower than background), whereas its expression level was at least two times background in CS and CS_5AL-10 CTRLs, and raised to seven times background in CS under SS. This sequence, expressed and regulated only in *T. aestivum*, is a typical example of a gene likely located on the D genome or regulated by genomic elements of D genome (see below).

Clusters 15 and 19 represent probe sets down-regulated by SS more in Creso than in CS (Figure 3B). Many probe sets from clusters 15 and 19 are related to genes encoding microtubules, cytoskeleton elements, cell wall biosynthetic enzymes and drought-responsive proteins. A common response to drought stress in plant is a block in cell growth to reduce transpiration leaf surface [42,43]. The repression of the genes involved in the synthesis of cytoskeleton and the production of enzymes involved in cell wall synthesis/degradation support the stress-dependent reduction in cell growth. The transcriptomic data suggest that Creso requires a higher stress level compared to CS to activate this response.

A peculiar expression behavior was found in the 91 probe sets grouped in Cluster 6 (Figure 3C). Their expression levels were significantly up-regulated in CS_5AL-10 exposed to MS only. 13 probe sets of this cluster show similarity with transposon and retrotransposon sequences.

Cluster 21 groups probe sets slightly up-regulated by drought stress in bread wheat samples only, although the expression level of these probe sets in CTRL conditions was higher in Creso than in CS (Figure 3D). Two SNF1-related protein kinases (*CIPK10*-CBL-interacting protein kinase, Ta.25609.1.S1_at and *CIPK9*, Ta.451.1.S1_at) are present in cluster 21. The homologous Arabidopsis gene, SNF1-related protein kinase 2 (*SnRK2*), encodes an osmotic stress-activated protein kinase. Insertional mutants exhibited drought hypersensitivity in roots and, conversely, transgenic plants over-expressing *SnRK2 *displayed a higher drought tolerance associated with the up-regulation of many stress-responsive genes as *RD29A*, *COR15A*, and *DREB1A/CBF3 *[44].

### Metabolic pathways activated under drought stress: conserved and divergent features

The Affymetrix GeneChip^® ^Wheat Genome Array covers all the genes corresponding to the main biosynthetic pathways. In many cases, each enzyme is represented by more than one probe set, apparently due to the existence of different isozymes and alleles. We took advantage from this feature to investigate the regulation of the enzymes involved in two main drought-related pathways leading to the accumulation of ABA and proline.

**Figure 4:**
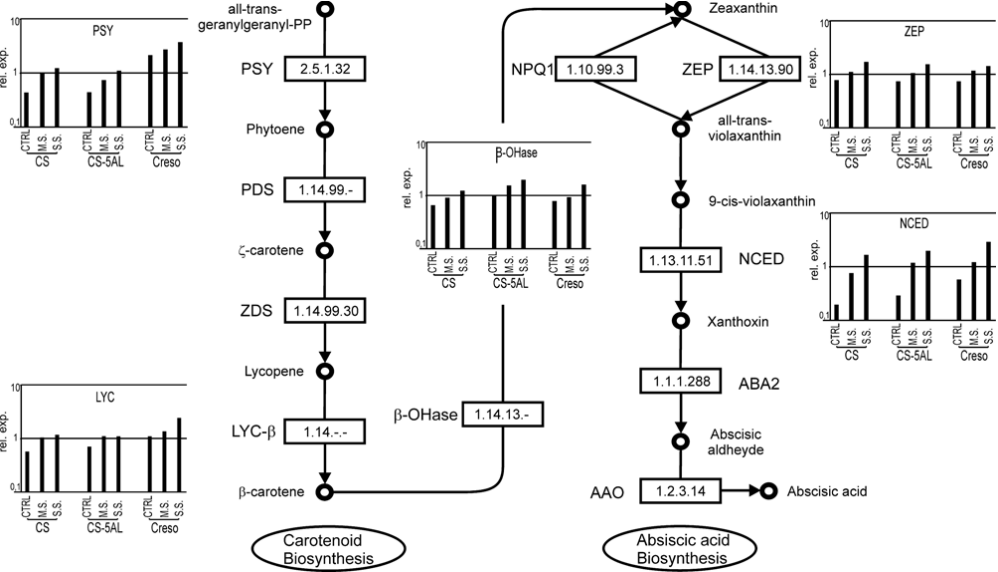
**Brief overview of the ABA pathway (inferred by KEGG, **[45]**)**. On the left side the β-carotene biosynthesis steps. On the right the ABA-dedicated enzymatic reactions. Several probe sets related to ABA synthesis enzymes (PSY, LYC- β, β-OHase, NCED) were up-regulated by drought stress. Their expression levels based on array data are showed in the corresponding histograms. 2.5.1.32 = Phytoene synthase (PSY); 1.14.99.-= Phytoene desaturase (PDS); 1.14.99.30 = ζ-carotene desaturase (ZDS); 1.14.-.-= Lycopene β-cyclase (LYC-β); 1.14.13.- = β-carotene hydroxylase (β-OHase); 1.10.99.3 = Violaxanthin de-epoxidase (NPQ1); 1.14.13.90 = Zeaxanthin epoxidase (ZEP); 1.13.11.51 = 9-*cis*-epoxycarotenoid dioxygenase (NCED); 1.1.1.288 = xanthoxin dehydrogenase (ABA2); 1.2.3.14 = Abscisic aldehyde oxidase (AAO).

Thirty-two probe sets related to ten enzymes of carotenoid-ABA biosynthesis are present on the Affymetrix wheat microarray. Figure 4 illustrates a schematic representation of the biosynthetic pathway [45,46] with indication of the probe sets corresponding to the enzymes modified during drought treatment. In the additional file 2 (ABA-related probe sets) all probe sets corresponding to ABA biosynthetic enzymes are listed with their expression level. The transcripts encoding *NCED *were the most significantly up-regulated in response to drought in all genotypes (Figure 4). In other species, a detailed study of *NCED *expression during water stress has shown a tight correlation between mRNA expression, protein level, and ABA content in dehydrated leaves and roots, indicating a regulatory role of NCED in ABA biosynthesis [47]. Furthermore, over-expression of *NCED *in tomato plants results in the over-production of ABA [48]. The microarray carries 12 *NCED-*related probe sets (additional file 2: ABA-related probe sets): four were not expressed, four were expressed and not modulated during dehydration treatment, while four probe sets (Ta.12813.1.S1_x_at, Ta.12813.2.S1_x_at, TaAffx.13292.1.S1_at and TaAffx.13292.1.S1_s_at) were differentially expressed with a cluster 3-related expression profile (the more severe the stress, the higher the expression level in all genotypes). These data support the hypothesis that, like in other plants, wheat ABA synthesis is regulated mainly through transcriptional induction of *NCED*.

In plants, the β-xanthophylls violaxanthin and neoxanthin are biosynthetic precursors of ABA [49]. In our experiment we have indeed found that several probe sets encoding enzymes involved in β-xanthophyll biosynthesis were up-regulated by drought to the same extent in all three genotypes (Figure 4). Ta.20776.1.S1_at, homologous to phytoene synthase 1 (*PSY1)*, was induced during dehydration with a cluster 1 type expression profile, a behavior different from what observed in maize leaves, where *PSY2*, rather than *PSY1 *is induced by water stress [50]. A probe set encoding lycopene β-cyclase (*LYC-β*; 3 probe sets on the microarray), a probe set encoding β-carotene hydroxylase (*β-OHase*; 3 probe sets on the microarray) and a probe set encoding zeaxanthin epoxidase (*ZEP*; 2 probe sets on the microarray) were all induced in response to drought suggesting a general up-regulation of the whole pathway. Nevertheless, whereas the expression level of the two probe sets related to *LYC-β *and *β-OHase *in CTRL and drought samples were significantly different, the *ZEP *probe set failed the statistical test even if it was induced by drought stress. Abscisic aldehyde oxidase 3 (AAO3) catalyzes the final step in abscisic acid biosynthesis in Arabidopsis [51]. One probe set with high sequence similarity with the *AAO *gene is carried by the wheat microarray though its expression was not modified under the tested conditions. This might suggest that in wheat *AAO *has a different regulation profile compared to Arabidopsis where this transcript is usually induced in response to drought stress [51].

**Figure 5:**
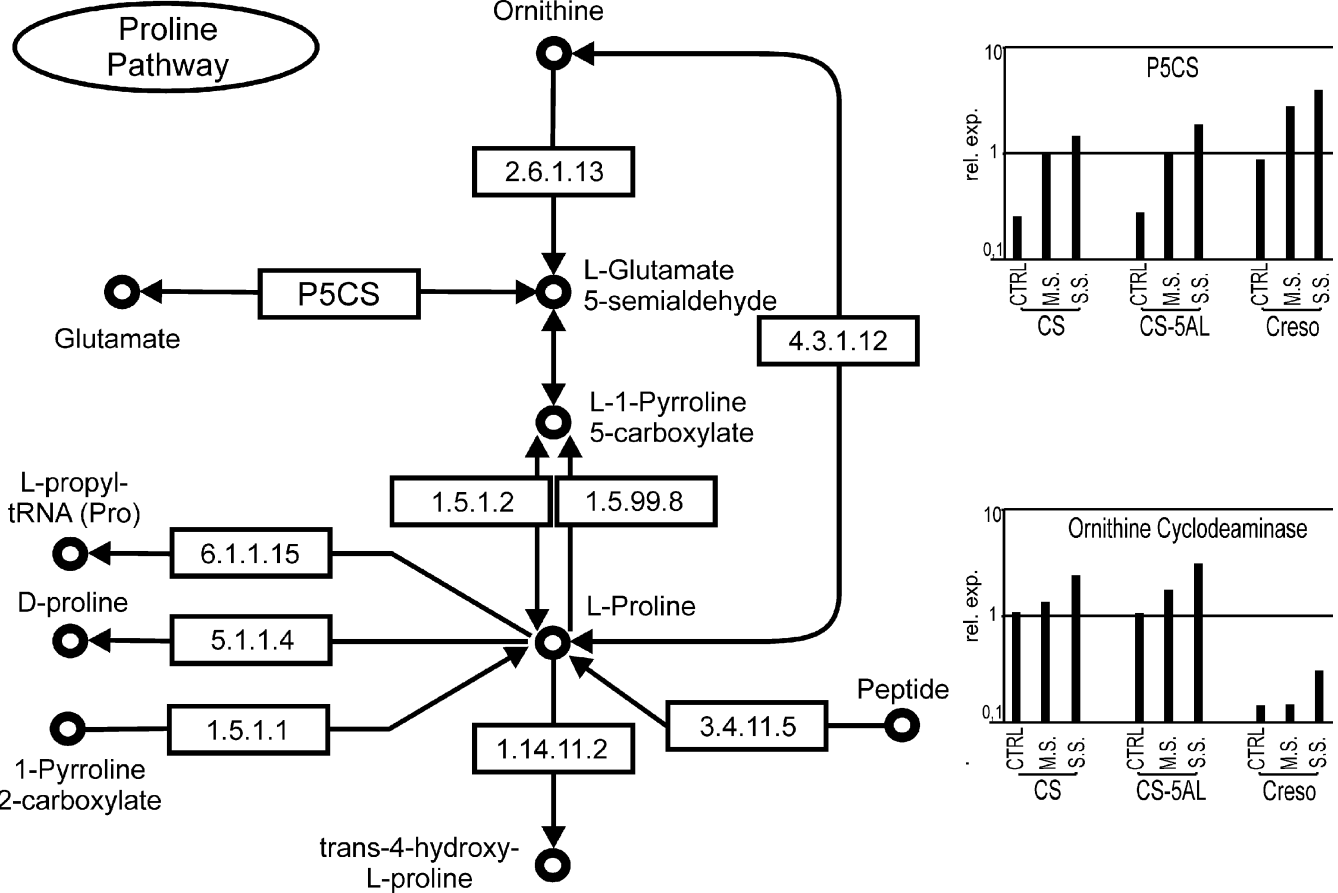
**Overview of proline biosynthesis and main catabolic reactions (inferred by KEGG**, [45]). Only the P5CS and ornithine cyclodeaminase probe sets were found to be differentially expressed. The expression levels based on array data are showed in the corresponding histograms. 2.6.1.13 = ornithine aminotransferase; P5CS = Δ-pyrroline-5-carboxylate synthase; 4.3.1.12 = ornithine cyclodeaminase; 1.5.1.2 = Δ-pyrroline-5-carboxylate reductase; 1.5.99.8 = proline dehydrogenase; 6.1.1.15 = prolyl-tRNA synthase; 5.1.1.4 = proline racemase; 1.14.11.2 = prolyl hydroxylase; 3.4.11.5 = proline iminopeptidase; 1.5.1.1 = pyrroline-2-carboxylate reductase.

In plants, proline can be synthesized starting from either glutamate or ornithine. Ornithine is the preferential precursor under normal conditions [52], whereas proline is made directly from glutamate under stress conditions [53]. The glutamate-dependent pathway begins with the conversion of glutamate to pyrroline-5-carboxylate catalysed by P5CS (Δ-Pyrroline-5-carboxylate synthase) [45,54,55] (Figure 5). Subsequently, the pyrroline-5-carboxylate is turned into proline by pyrroline-5-carboxylate reductase (P5CR). *P5CS *is represented by one probe set (Ta.7091.1.S1_at) (see additional file 3: Proline-related probe sets) and its expression level was induced along with the drought stress intensity in all genotypes. The expression level in Creso was constantly higher than in CS and CS_5AL-10 samples (Figure 5). P5CS is considered the main regulatory enzyme in proline synthesis [56] and its up-regulation in stress conditions supports proline production also in wheat plants. Three probe sets showed high sequence similarity with *P5CR*, though no one was differentially expressed.

**Figure 6:**
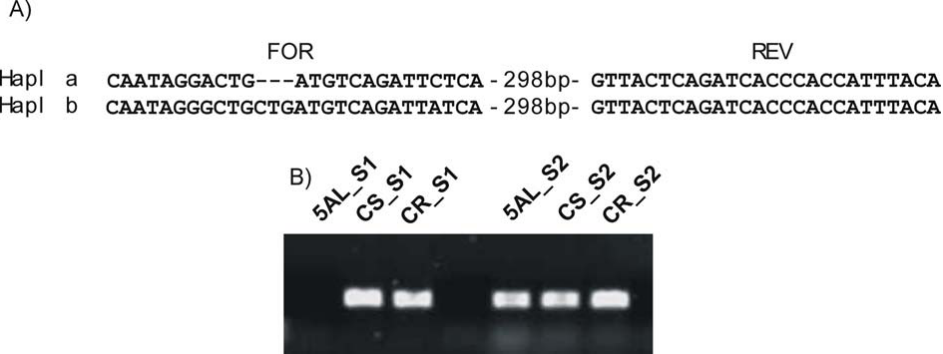
**PCR-mapping of genes putatively located on the 5AL-10 deleted region**. The example reported refers to the sequence corresponding to probe set Ta.9404.1.A1_at. The sequence is moderately similar to a rice kinase (Os03g0107400). A) Alignment between the sequence used to design the probe set (Hapl a) and a homoeologous gene (Hapl b). Sequences used to design the primers are highlighted. The forward (FOR) primer is haplotype-specific, while the reverse (REV) primer is common. B) PCR reaction performed using the primers shown in A). Since the primer pair for Hapl a amplified the target sequence in both CS and Creso and no amplicon was observed in CS-5AL-10, the haplotype ''a'' correspond to a sequence located on to the 5AL-10 deleted region. 5AL: CS-5AL-10 deletion line. CS: Chinese Spring. CR: Creso; S1: Haplotype 1 – specific primers; S2: Haplotype 2 – specific primers.

The synthesis of proline via the ornithine-dependent pathway is regulated by ornithine-δ-aminotransferase (OAT). Five *OAT*-related probe sets were present on the microarray, and none of them was differentially expressed (see additional file 3: Proline-related probe sets). Alternatively, ornithine cyclodeaminase is able to convert ornithine into proline in a one step reaction [41]. The ornithine cyclodeaminase-related probe set (TaAffx.3441.1.S1_at) was induced by drought stress in CS and CS_5AL-10 (Figure 5), while its expression level in Creso was lower than background in CTRL and MS and minimally induced in SS. No other probe sets corresponding to enzymes of proline pathway or proline catabolism were modified in the drought treated samples.

Transcriptional analysis of the genes involved in proline metabolism highlighted that all genotypes activate the transcription of *P5CS*, although its expression level was higher in Creso than in bread wheat. On the contrary, ornithine cyclodeaminase was preferentially expressed and up-regulated by drought in bread wheat (Figure 5). The list of probe sets related to proline metabolism and the corresponding expression levels are summarized in the additional file 3: Proline-related probe sets.

### Expression level-based gene mapping

A specific absence of gene expression observed in durum wheat (AABB) compared to bread wheat (AABBDD) could be due to the localization of the corresponding genes on D genome. Similarly, a specific absence of gene expression in the CS_5AL-10 deletion line, could be due to the localization of the corresponding genes in the deleted chromosomal region. The transcriptomic data allowed the identification of genes whose expression level is very low ("absent call") in all Creso or CS_5AL-10 samples and high in all CS samples. Using the very stringent parameters described in Materials and Methods with the expression level in the "present call" more than 3 times the background value, 278 genes putatively located on the D genome and 28 genes putatively located on the long arm of chromosome 5A were identified. Using more relaxed criteria with a threshold of 1 time the background value and no filter on "present calls", the analysis yielded 1049 genes putatively located on the D genome and 127 genes putatively located on the long arm of chromosome 5A.

To verify this mapping assignment, a collection of randomly selected probe sets corresponding to genes putatively localized on the D genome and all probe sets putatively localized on the 5AL-10 deleted region, were mapped as described in Materials and Methods. Briefly, oligonucleotides able to discriminate between haplotypes were designed (Figure 6) and used to amplify DNA from the 3 genotypes (Creso, CS and CS_5AL-10). The obtained PCR bands were then re-sequenced in order to confirm their correspondence to the targeted haplotype.

Seventy genes putatively mapping on the D genome were analyzed. Of these, 38 gave a useful mapping result and for 32 genes, haplotype "a" (which was the one showing maximum homology to the Affymetrix probe set) was found to map on genome D. Similarly, 25 genes, putatively located on the CS_5AL-10 deleted region, were analyzed. 13 gave a specific mapping result and for 12 of them, haplotype "a" was found to map on CS_5AL-10, according to expectations. Only one exception was found (Ta.3072.1.S1_at), with haplotype "b" mapping on CS_5AL-10, and haplotype "a" on all genomes. The probability of these results occurring by chance is less than 0.001 according to the χ^2 ^test for both datasets, suggesting that the criteria used to predict the chromosome/genome location based on expression data were highly reliable. Since the identification of the genes putatively mapped on D genome (or on CS_5AL-10) was based on expression data, the sequences not confirmed by mapping experiment probably represent genes whose expression is controlled by factor(s) carried on D genome (or on CS_5AL-10). The failure of several gene localizations could also be explained considering that the wheat genome is far from being completely sequenced; consequently a non-specific primer design is likely to occur.

The detailed results for all probe sets tested are reported in the additional file 4: D genome expression based mapping and in the additional file 5: 5AL-10 expression based mapping.

Some of the genes defined as located on the D genome (relaxed threshold), were also up-regulated by drought stress in CS. Among them there are four aquaporins, an aldose reductase, a bZIP transcription factor and a dehydrin (cluster 2 Figure 3A). Although these genes might have an effect on drought tolerance, it is unlikely that they are responsible for inter-specific differences in drought tolerance between durum and bread wheat.

Among the probe sets putatively localized on chromosome 5A (relaxed threshold) there are two well known stress-related genes: the dehydrin *WCOR719 *and *CBF1*, a member of the *CBF *cluster involved in cold-tolerance [57]. The *CBF *locus has been precisely mapped on long arm of the chromosome 5A and represents a marker for many loci controlling traits such as cold tolerance, and amino acid content in response to salt stress [58-60] all absent in CS_5A-10. Three probe sets (Lipid Transfer Protein, glioxylase and PSI-related protein) putatively located on long arm of chromosome 5A were also up-regulated in drought stressed CS.

## Discussion

Changes in mRNA expression following abiotic stresses have been extensively analyzed in plant species using microarrays. Different stress conditions, tissues and plant species, from Arabidopsis to cereal crops [10-17], have been investigated with microarray tools to find drought-regulated genes. Roots and leaves from seedlings were analyzed in wheat, barley and rice to describe the variation in gene expression induced in response to a dehydration shock imposed for few hours [16,61,62]. A slow-drying treatment was applied to study the transcriptome changes in wheat leaves at booting stage [63], or in developing kernels in maize and rice [64,65]. The comparative analysis of these data highlight that the conservation of the molecular response to dehydration across species and across experiments is generally low despite the presence of common regulatory mechanisms. For instance, the comparison between the genes found to be up- or down-regulated in Arabidopsis in response to dehydration by Matsui et al. [10] (more than 4,000 genes) with those found in the experiment here described highlighted only 68 and 180 common genes for CS and Creso, respectively. When Talamè et al. [17] compared the expression changes in leaves of barley plants subjected to slow or rapid drying, only a small portion of differentially expressed transcripts (about 10%) showed similar expression profile regardless of the dynamics of the water stress treatment. Variations in the response to drought depending on stress dynamics and on the stage of development were also reported for specific stress-responsive genes in durum wheat by De Leonardis et al. [66]. These results underline the importance of selecting stress conditions and tissues representing a physiological status that has a relevant role during field growth to identify pathways with a field relevant role in stress tolerance. In the present work, bread and durum wheat plants were subjected to a slow drought stress during grain filling, a critical stage for yield determination. The expression analysis was carried out on glumes, the last green and photosynthetically active tissues during grain filling. For these reasons our work, more than others, should give a close representation of a yield-relevant drought response.

The durum and the bread wheat genotypes considered in this work showed different reactions to the water stress treatment when grown in soils with the same amount of available water. CS and CS_5AL-10 were characterized by less negative leaf water potential values and they took much longer than durum wheat to reach these values, suggesting that a moderate water stress can already induce in these genotypes a response leading to a lower water loss. Differences in response to water stress between hexaploid and tetraploid genotypes were already described in previous reports. Gavuzzi et al. [67] compared 6 bread wheat, 6 durum wheat and 6 barley genotypes for physiological parameters following water stress, and found that bread wheat had the smallest water loss values. In a similar experiment, two hexaploid genotypes exhibited a higher level of proline with respect to two tetraploids in response to drought [68]. In our experiment, bread and durum wheat were characterized by a significantly different drought response. 106 probe sets were above the induction threshold when MS and CTRL samples were compared in CS. On the contrary, in Creso no probe set was above the induction threshold in the same comparison and only in SS *vs *MS and SS *vs *CTRL comparisons was possible to identify significantly induced/repressed genes. For instance, a set of genes encoding microtubule subunits and cell wall degradation enzymes was found down-regulated in Creso after the SS only (Figure 3B, clusters. 15 and 19). These transcriptional changes suggest a block in cell division and/or elongation supporting a smaller transpiring surface, a typical component of the plant drought response [42,43]. These observations indicate that the ability of CS to maintain a higher water potential during drought stress is associated to a more prompt molecular response, while Creso needs a more severe drought stress to activate any transcriptional response.

Although durum wheat and bread wheat are two distinct species with a different genome organization (tetraploid -AABB, and hexaploid -AABBDD, respectively), their share the same A and B genomes. The similarity between bread and durum wheat for sequences carried on A and B genomes is very high. Chantret et al. [69], studying the *Hardeness *locus (GenBank accession number AY491681, ca 100 Kb) in the A and B genomes of durum and bread wheat, highlighted that the two species share 97% and 99% of nucleotide identity for A and B genome, respectively. Furthermore, in a preliminary bioinformatic experiment, 104 randomly selected durum wheat ESTs (48,530 bp in total) were blasted to find the corresponding bread wheat sequences. Considering the BLAST best matches of all queries a mean identity of 98.5% (SD 0.02%) and a mean coverage of 95.2% were calculated, a results in agreement with the data of Chantret et al. The high level of genome identity between bread and durum wheat sustains the use of the same microarray for comparison of the transcriptomes of the two species, although the estimated 2% of sequence polymorphisms might lead to a small over-estimation of the transcriptomic differences.

In well-irrigated conditions, the CS (hexaploid wheat) and Creso (tetraploid wheat) transcriptomes were very different. About 6.6 thousand genes were found to be differentially expressed between the two wheat species (Table 2) on a total amount of approximately 30,000 expressed genes detected in bread wheat. Although about 80% of the genes expressed in absence of stress were common between Creso and CS, the drought response in the two genotypes was significantly different. 1,470 genes were above the threshold in the SS *vs *CTRL in Creso, while only 842 were detected in the same comparison in CS (Table 2) and among these genes only 201 were in common.

The analysis of the molecular response to drought revealed both common and genotype-specific features. A set of 556 genes were clustered in groups showing a very similar expression profile in Creso and CS (Figure 2A and 2B). Notably, many genes involved in well known drought-responsive pathways (i.e. ABA, proline, sorbitol and glycine-betaine) were commonly activated in all genotypes. The expression levels of the *NCED*-related probe sets, the key enzyme of ABA biosynthesis [35], were strongly up-regulated by water stress in all genotypes suggesting that the drought treatment imposed during the experiment entailed the activation of the ABA synthesis. In Arabidopsis, the accumulation of ABA in response to water deficit leads to the induction of the transcription factor *At-HB7 *(homeobox-leucine zipper) that, in turn, activates the expression of *At-RD20 *[70-72]. The probe sets coding for *HB7 *and *RD20 *wheat homologous sequences (TaAffx.108538.1.S1_at, Ta.9830.2.1.S1_at, respectively) were co-regulated with *NCED *and grouped in cluster 3 and 14, respectively, suggesting that this specific response is conserved in wheat.

Other genes grouped in commonly up- or down-regulated clusters play a role in primary metabolism, energy regulation, cell rescue or interaction with environment.

On the contrary, evidences for drought-responsive features associated to the different genomic structure of Creso, CS and CS_5A-10 were also present. Some drought-related genes were expressed at lower level (or not expressed) in Creso or in CS_5A-10 compared to CS (see clusters 2, 4 and, to less extent, 21), this finding can, to some extent, be associated to the absence of the D genome (or 5AL-10). Consequently, these genes could be located on the D genome (or 5AL-10), as demonstrated for some of them by expression based mapping, or could be controlled by genetic factors located on the D genome (or 5AL-10). Furthermore, several clusters were characterized by a higher expression level in Creso or in CS_5A-10 than in CS (see clusters 5 and 6), underlining that the different genome organization have a direct consequence on plant adaptation to stress. The 5AL-10 deleted region carries the *Cbf *cluster [59,60]. Although originally described as cold-regulated, the *Cbf *transcription factors are also induced during exposure to drought in wheat [66]. The absence of an important class of stress-related transcription factors can lead to modifications in the expression of many other genes located overall the genome. Furthermore, the analysis of the genes involved in the proline biosynthetic pathway have suggested an up-regulation of the ornithine-dependent pathway in bread wheat (Figure 5), while the enhanced expression of a set of transposons and retrotransposons was detected in CS_5AL-10 only. It is known that transposon and retrotransposon expression can be activated by biotic/abiotic stresses [73], our data suggest that the combination of abiotic stress with a "genetic stress" due to chromosomal deletion represent a suitable condition for a general up-regulation of transposon and retrotransposon-related mRNAs.

## Conclusion

Bread and durum wheat genotypes were characterized by different physiological reactions to the applied drought stress and by clearly different molecular responses. A moderate stress was sufficient to produce a significant change in expression level of hundreds of transcripts in CS and CS_5AL-10, while only a severe water stress could produce a similar molecular response in Creso, suggesting that CS and CS_5AL-10 activated protection mechanisms faster and more efficiently than Creso. The genome organization accounted for differences in the expression level of hundreds of genes located on D genome or controlled by regulators located on the D genome. When a genomic stress (deletion of a chromosomal region) was combined with the low water availability, a molecular response based on the activation of transposons and retrotransposons was observed.

## Methods

### Experimental design

To provide a global study of transcriptome changes under drought stress, the gene expression of a durum wheat genotype (*Triticum durum *Desf. cultivar Creso) and of two bread wheat genotypes (*Triticum aestivum *L. cultivar Chinese Spring -CS- and its deletion line CS_5AL-10) were investigated. The 5A chromosome deletion line (5AL-10) developed by Endo and Gill [74] lacks the distal part (43%) of the long arm of chromosome 5A, the breaking point being in band L1.6 . The deleted region contains several loci involved in freezing tolerance [27], ABA and osmoprotectant accumulation [29].

Each genotype was subjected to two different levels of water stress at the grain filling stage. After anthesis, three different levels of soil water content (SWC) were induced as described below: control (CTRL; SWC = 28%), moderate stress (MS; SWC = 18%), and severe stress (SS; SWC = 12.5%). SWC was calculated as the percentage of water with respect to the total fresh weight of the soil. For each sample, three biological replicates were performed, for a total of 27 hybridizations.

### Drought stress

The durum wheat genotype and the two bread wheat genotypes were sown in pots (16 × 16 cm) on a mixture of soil, sand and peat (6:3:1) in a growth chamber with controlled temperature, humidity and photoperiod. Five plants per pot were grown at 10°C day/7°C night, 60% relative humidity, 12 h light:12 h darkness, 500 μmol m^-2 ^s^-1 ^photon flux density until the third leaf stage then 22°C day/18°C night, 55% relative humidity, 16 h light:8 h darkness 500 μmol m^-2 ^s^-1 ^photon flux density until harvesting of the samples.

Soil water content was maintained close to field capacity (28%) until plants reached the stage of 3 days post anthesis (3DPA), when watering was stopped. The water status of the plants was monitored by measuring the water potential with a pressure chamber (PMS Instrument Co., Corvallis, OR, USA). The control plants (CTRL) continued to be watered while the soil of water stressed plants was allowed to dry until 18% of SWC (mild stress or MS) and 12.5% of SWC (severe stress or SS). SWC was monitored daily by checking the weight of the pots. The weight corresponding to the two levels of stress (18 and 12.5% SWC) were calculated by also considering the difference in biomass between genotypes. The biomass was evaluated in control conditions. In all genotypes, two and three days were required to reach 18 and 12.5% SWC values, respectively. After that, the reached values of SWC were maintained weighting of the pots and adding the needed amount of water twice a day (early morning and late afternoon). The samples were harvested at noon at 9DPA (Creso) and 11DPA (CS and CS_5AL-10), when all genotypes reached a comparable level of stress, as estimated by leaf water potential. These stress conditions resulted in flag leaf water potentials of -2.1/-2.6 MPa and -3.3/-3.9 MPa in MS and SS, respectively (Table 1). Glumes and flag leaf tissues were sampled and immediately frozen in liquid nitrogen.

### RNA isolation and array hybridization

RNA was extracted using the TRIZOL reagent according to the method published by the Arabidopsis Functional Genomics Consortium  and further cleaned using RNeasy columns according to the Qiagen RNeasy Mini Handbook. RNA was quantified and quality assessed by running several dilutions of each sample using the Agilent RNA 6000 nano Kit and Agilent Bioanalyzer 2100.

RNA samples were processed following the Affymetrix GeneChip Expression Analysis Technical Manual (Affymetrix, Inc., Santa Clara, CA). Single-stranded, then double-stranded cDNAs were synthesized from the poly(A) mRNA isolated from 5 μg of total RNA for each sample using the Affymetrix One-Cycle Labeling kit and Control reagents. The resulting *ds*-cDNA was column-purified and then used as a template to generate biotin-tagged cRNA from an *in vitro *transcription reaction (IVT), using the Affymetrix GeneChip IVT Labelling Kit. Fifteen μg of the resulting biotin-tagged cRNA was fragmented to strands of 35–200 bases in length following prescribed protocols (Affymetrix GeneChip Expression Analysis Technical Manual) and then hybridized at 45°C with rotation for 16 h (Affymetrix GeneChip Hybridization Oven 640) to probe sets present on an Affymetrix GeneChip^® ^Wheat Genome Array. The arrays were washed and then stained (SAPE, Streptavidin-phycoerythrin) on an Affymetrix Fluidics Station 450 followed by scanning with a GeneChip Scanner 3000. Wheat microarray design and expression profiling data are available in PlexDB  as experiment TA23: 'Drought stress in Wheat at grain filling stage'.

### Data processing and analysis

GeneChip^® ^hybridization quality was ensured using the standard Affymetrix controls. B2 oligonucleotides were spiked into each hybridization cocktail. PolyA controls (*lys, phe, thr, dap*) and hybridization controls (*BioB, BioC, BioD and Cre*) were used to monitor the labeling and hybridization processes.

Raw intensity values were normalized by RMA (Robust Multi-array Average) [75] using the R package Affymetrix library [76]. The same library was used to run the MAS 5.0 algorithm on raw data to produce a detection call for each probe set. These detection calls ("present", "marginal" or "absent") were used to apply an initial filtering step, since genes not expressed ("absent") represent experimental noise and can generate false positives. We removed from analysis all the probe sets that didn't show all the three "present" calls in at least one sample. R-squared linear correlation coefficients were computed on the RMA expression values (log2-transformed) for each set of biological triplicates.

RMA filtered data were imported to the software Genespring GX 7.3 (Agilent Technologies, Santa Clara CA) and all subsequent analyses were carried out with this software. Three comparisons for each of three genotypes were done: MS *Vs *CTRL, SS *Vs *CTRL and SS *Vs *MS. Differentially expressed probe sets were identified through a Welch t-test with Benjamini and Hochberg false discovery rate correction for multiple tests [31]. Differences in gene expression were considered to be significant when p-value was lower than 0.05 and induction or repression ratio was equal or higher than 2-fold. Principal Component Analysis (PCA,[77]) was then employed to assess the role of genotype and stress treatment in the explanation of the variation in the dataset.

Clusters of genes with distinctive expression patterns were searched with QT (Quality Threshold) cluster analysis [33]. QT clustering algorithm groups genes into high quality clusters based on two parameters: "minimum cluster size" and "minimum correlation". The minimum cluster size was set to 30 and minimum correlation to 0.75. Functional gene categories over-represented in the clusters in comparison with the whole microarray were searched at the MIPS *Arabidopsis thaliana *database (MAtDB) Functional Catalogue (FunCat) . MIPS FunCat is a hierarchical database that links Arabidopsis locus identifiers to functional categories. The FunCat database currently contains 28 main categories subdivided into 1289 subcategories [34]. Blast searches were done using HarvEST: Affymetrix Wheat1 Chip 1.50 , and only the annotations of wheat probe sets with a homology level cut-off equal or lower than E-value = e^-10 ^were considered.

### q-RT-PCR and identification of reference genes

Three μg of total RNA of each sample were reverse transcribed using oligo (dT)_18 _primer with M_MLV Reverse Transcription Reagents (Promega) according to the manufacturer's standard protocol. The reaction was incubated at 40°C for 10 min, then 45°C for 50 min. The RT was heat-inactivated at 70°C for 15 min. Subsequently, the cDNAs were quantified using a Qbit™ fluorometer (Invitrogen), diluted and used for q-PCR amplifications with specific primers.

q-RT-PCR was performed with SYBR Green fluorescence detection in a qPCR thermal cycler (ABI PRISM 7300, Applied Biosystems). Each reaction was prepared using 5 μl from a 0.2 ng/μL dilution of cDNA derived from the RT reaction, 10 μl of SYBR Green PCR Master Mix (Applied Biosystems), 0.5 μM forward and reverse primers, in a total volume of 25 μl. The cycling conditions were: 10 min at 95°C, followed by 40 cycles of 95°C for 15 sec and 60°C for 1 min. Melting curve analysis was performed to evaluate the presence of non-specific PCR products and primer dimers. The q-PCR data were plotted as the ΔRn fluorescence signal versus the cycle number. The ABI PRISM 7300 Sequence Detection System software calculates the ΔRn using the equation ΔRn = (Rn+) - (Rn-), where Rn+ is the fluorescence signal of the product at any given time and Rn- is the fluorescence signal of the baseline emission during cycles 6–13. An arbitrary threshold was set at the midpoint of the log ΔRn versus cycle number at which the ΔRn crosses the threshold. To calculate the fold changes (FC) we used the following formula: FC = 2^-ΔΔCT where: ΔΔCT = (CT_target gene _- CT_reference gene_)_treatment _- (CT_target gene _- CT_reference gene_)_control_. The CT data are expressed as average of three experimental replicates. qRT-PCR data were compared to the corresponding microarray expression values by mean of Pearson product-moment correlation coefficients.

Transcripts of stably expressed genes are crucial internal references for gene expression data normalization. To search for a gene(s) with stable level of expression in the conditions used in the present work the probe sets showing a "present" call in all hybridization examined based on the MAS 5.0 algorithm, were considered. Among all probe sets on the wheat microarray, 17,134 were called "present" in all samples and 4,768 probe sets showed a level of expression, normalized to the corresponding probe set median, between 0.66 and 1.5 indicating that the corresponding mRNA showed less than 1 fold change variation in their expression across all samples. These probe sets were then listed according to their Coefficient of Variation (CV = standard deviation mean^-1^). The best three ranking probe sets based on CV and expression level were: Ta.1532.1.S1_a_at, a probe set annotated as translation initiation factor (CV 0.052), Ta.4093.1.S1_at encoding for a spastin-like protein (CV 0.055) and Ta.24299.1.S1_at encoding for polyubiquitin (CV 0.063). The stability of their expression across wheat samples was further checked by qRT-PCR and polyubiquitin was selected as reference gene.

### Identification and validation of genes putatively located on the D genome or on the deleted region of CS_5AL-10

Since the genomes of durum wheat (AABB) and of the deletion line (AA^5AL10^BBDD) represent a portion of the complete bread wheat genome (AABBDD) we run a bioinformatic experiment to compare the transcriptome of the three genotypes to find genes putatively located on genome D or 5A deleted region. We took advantage of the MAS 5.0 detection algorithm that allows to discriminate probe sets corresponding to mRNA reliably detected (present) from those not reliably detected (absent) in the samples. A probe set was considered putatively located on genome D if it matched the following conditions: i) all "absent" calls in the 9 Creso replicates and expression values lower than background value; ii) all "present" calls in the 18 CS and CS_5AL-10 replicates and expression values higher than 3 times the background value. The same approach was applied to search genes putatively located on long arm of chromosome 5A. In this case the microarray data relative to deletion line and CS were used.

The putative map positions of a selected group of probe sets matching these conditions were subjected to PCR validation on genomic DNA. The sequences used to construct the Affymetrix probe set were downloaded from the GrainGenes database . Then, homoeologous wheat transcripts were sought using Blast and downloaded from . The sequence used by Affymetrix to design the probe set was designed as haplotype "a" and subsequently each haplotype was labeled progressively on the basis of the sequence similarity. The various haplotypes were aligned to each other using Clustal W, and oligonucleotides able to discriminate between haplotypes were designed. These oligonucleotides were then used to amplify DNA from the 3 genotypes (Creso, Chinese Spring and CS_5AL-10). The obtained PCR bands were then re-sequenced in order to confirm that they corresponded to the targeted haplotype. 25 genes putatively mapping on 5AL and 70 genes putatively mapping on D genome were analyzed.

## Authors' contributions

AA interpreted the microarray results. AA and FF carried out the bioinformatic analyses. AMD assisted with material isolation from drought stressed plants. ER carried out qRT-PCR and the searching analysis of internal reference genes. LT and GGiu carried out the expression level-based gene mapping. AMM, GGal, GGiu and LC participated in the design of the study. LC coordinated the study. LD linked expression data to physiological drought effects. AA, AMM, and LC wrote the manuscript. All authors read and approved the final manuscript.

## Supplementary Material

Additional file 1 **All differentially expressed genes in drought stress comparisons**. The list of the 3,056 differentially expressed genes in drought comparisons is sorted in column A. For each probe set are reported the relative QT-cluster, the CTRL (control samples) expression levels in the three genotypes and the expression ratio of each comparison. Yellow boxes highlight differentially expressed (DE) probe sets. In X and Y columns the AGI code and Arabidopsis annotations of the homologous Affymetrix wheat probe set. The last column reports the list of MIPS functional categories (Ruepp et al. 2004) associated to the each gene.. Click here for file

Additional file 2 **ABA pathway-related probe sets on Affymetrix Wheat Genome Array^®^**. The probe sets related to the ABA and β-carotene pathways are reported in additional file 2. Grey rows highlight differentially expressed probe sets. CS = Chinese Spring, CS-5AL = Chinese Spring 5A deletion line, CTRL = control condition, MS = mild stress condition, SS = severe stress condition. Click here for file

Additional file 3 **Proline pathway-related probe sets on Affymetrix Wheat Genome Array^®^**. The probe sets related to proline pathway are reported in additional file 3. Grey rows highlight differentially expressed probe sets. CS = Chinese Spring, CS-5AL = Chinese Spring 5A deletion line, CTRL = control condition, MS = mild stress condition, SS = severe stress condition. Click here for file

Additional file 4 **D genome expression based mapping**. 70 genes putatively mapping on the D genome were mapped by PCR. Of these, 38 gave a specific mapping result and for 32 genes, haplotype a) was found to map on genome D. 6 deviations were found, with different haplotypes mapping on the D genome, and haplotype a) on all genomes. Click here for file

Additional file 5 **5AL-10 expression based mapping**. 25 genes putatively located on the CS_5AL-10 deleted region were mapped by PCR. 13 gave a specific mapping result and for 12 of them, haplotype a) was found to map on CS_5AL-10, according to expectations. Click here for file
